# Social Integration in Higher Education and Development of Intrinsic Motivation: A Latent Transition Analysis

**DOI:** 10.3389/fpsyg.2022.877072

**Published:** 2022-06-14

**Authors:** Marion Reindl, Tanja Auer, Burkhard Gniewosz

**Affiliations:** Department of Educational Science, University of Salzburg, Salzburg, Austria

**Keywords:** social integration, intrinsic motivation, higher education, students, latent transition analysis

## Abstract

This study, based on the self-determination theory, investigates the link between university students' social peer and teacher integration and intrinsic motivation development. Both integration contexts are expected to contribute to the student's development, either additive or compensatory. The analyses rely on a nationally representative sample of 7,619 German university students (NEPS data set) and cover the time between the 3rd and 5th semesters in a longitudinal design. Person-centered analytical tools were applied to tap interindividual differences in the motivational trajectories as well as in integration profiles. Latent transition analyses revealed distinct links between the motivational trajectories (*Increase* [*n* = 532], *Moderate Decrease* [*n* = 2580], *Decrease* [*n* = 4,507]) and the integration profiles (*Highly Integrated* [*n* = 2,492], *Moderately Integrated* [*n* = 3832], *Isolated* [*n* = 1,144], *Peer Deprivated* [*n* = 151]), pointing to additive effects of teacher and peer integration. Positive trajectories were more likely in the *Highly* than in *Moderately Integrated* profiles. The two profiles pointing to below-average integration levels (*Isolated* and *Peer Deprivated*) showed the same probabilities for rather negative trajectories. The results are discussed against the backdrop of self-determination theory and additive vs. compensatory effects of teacher and peer integration, proposing a threshold model.

## Introduction

University students' intrinsic motivation is vital to a student's identity (La Guardia, 2009), academic achievement (Richardson et al., 2012; Taylor et al., 2014; Tasgin and Coskun, 2018), and university retention (Morrow and Ackermann, 2012). Against this background, it is quite problematic that empirical research revealed that students' intrinsic motivation declined during their bachelor's studies (Pan and Gauvain, 2012). However, results showed that although the intrinsic motivation of most students decreased, some students showed favorable developmental trajectories (stability or an increase on a quite high level; Gillet et al., 2017; Corpus et al., 2020) and show, therefore, a lower risk for university dropout (Rump et al., 2017; McDermott et al., 2019).

For the explanation of developmental trajectories of intrinsic motivation, the self-determination theory highlights a good integration into important socialization contexts as a protective factor (Ryan and Deci, 2000a). At the university, peers (fellow students) and teachers (Tinto, 1975; Astin, 1993; Casanova et al., 2021) are highlighted as two important socialization contexts. Previous studies provide evidence that both contexts jointly influence the development of intrinsic motivation (Wentzel, 1998; Shin and Bolkan, 2021). However, a more accurate understanding of the effects of social integration can be achieved if both relationships are investigated simultaneously (Jager, 2011). At least, two patterns can be expected: (1) Students who are integrated into both contexts on a comparable level (convergent integration) and (2) Students who are only integrated into one context successfully (divergent integration). This allows for an investigation of additive as well as compensatory effects on the development of intrinsic motivation. Above and beyond the investigation of additive effects (the more the better), the focus on compensatory effects within divergent integration profiles might enhance our understanding if a high integration into one context might compensate for a low integration into the other context, and in turn, might increase the possibility for a favorable development of intrinsic motivation.

Taken together, this study aims to examine developmental trajectories of intrinsic motivation (e.g., decrease, stability, increase), differential integration profiles (e.g., convergent and divergent) across contexts (peers and teachers), and how both are related (e.g., additive or compensatory effects), by applying person-centered methods. This allows a more holistic-interactionistic perspective on the developmental process of the individuals by directly addressing between-person idiosyncrasies. Rather than focusing on an overall result pattern, person-centered analyses aim at identifying homogeneous subsets of individuals within the heterogeneity of a sample (e.g., Laursen and Hoff, 2006; Xie et al., 2020).

### Intrinsic Motivation: Definition and Development

The self-determination theory describes that intrinsic motivation concerns actions that are interesting and joyful for their own sake (Ryan and Deci, 2000a). A person who is intrinsically motivated initiates actions for their inherent value (e.g., satisfaction, enjoyment) and not for some consequences (e.g., external rewards). Results of variable-centered studies showed a quite ambivalent picture of developmental trajectories for intrinsic motivation. Some studies reported that the intrinsic motivation of university students decreases during bachelor's studies (Brahm and Gebhardt, 2011; Pan and Gauvain, 2012) but there is also evidence that the motivation remains relatively stable (Müller and Palekčić, 2005; Bieg et al., 2017). A person-centered study on the development of intrinsic motivation found three groups of developmental trajectories of intrinsic motivation. Students were grouped into a decline profile, moderate increase profile, and strong increase profile (Ratelle et al., 2004). Another study by Gillet et al. (2017) investigated profiles of autonomous (including intrinsic motivation) and controlled motivation during one semester and if students changed their profile. The results showed that motivational orientations (highly autonomous/ or highly controlled) are quite stable. However, students on moderate levels (autonomous or controlled), were more likely to change their motivational orientations, indicating that students might also be able to increase their autonomous motivation over time. Taken together, besides a decrease or stability of most of the students' intrinsic motivation, some students showed a favorable development (increase). Individual characteristics, such as age and gender, were shown to weakly predict individual intrinsic motivation pathways (Gillet et al., 2009). However, further explanations for individual pathways of intrinsic motivation might be found in the social environments of students, such as teachers and peers, e.g., fellow students (Tinto, 1975; Astin, 1993).

### Social Integration at the University: Teachers and Peers

Social integration is defined as students' perceptions of interactions, e.g., with the peer group and teachers (Tinto, 1993; Wolf-Wendel et al., 2009; Suhlmann et al., 2018; Shin and Johnson, 2021). Interactions with teachers may be characterized as more hierarchical, for example, showing interest in their students. Fellow students as non-hierarchical interaction partners may provide opportunities to discuss topics on eye level as well as for joint activities. Research has shown that, especially the informal aspects of the teacher integration, such as interactions referring to personal aspects, and the formal aspects of peer interactions, such as interactions regarding study-related aspects, showed positive effects on learning outcomes, such as achievement (Severiens and Schmidt, 2008).

Based on attachment theory (Bretherton, 1995), the level of integration into one context should be positively related to the level of integration into another context (convergent profiles; Ciarrochi et al., 2017). Although researchers primarily focused on adolescents and how they feel supported by teachers and peers, results revealed that most of the students reported convergent integration profiles, i.e., the support of both contexts is high, medium, or low (Ciarrochi et al., 2017). Reasons can be found in personal characteristics, such as self-esteem (Schaeper, 2020) or an open-mindedness toward new people (Buote et al., 2007), that might generally help to establish good relationships with various contexts, e.g., teachers and peers. However, there might also be the possibility that the level of integration differs between the two contexts (divergent profiles). For example, Ciarrochi et al. (2017) reported a profile in which students feel highly supported by their peers but not by their teachers. In addition, a study by Rosenfeld et al. (2000) reported a profile of students who felt supported either by their peers or by their teachers. Explanations might be found in the organizational structure of the universities as well as the expectations of the persons themselves. In detail, departmentalized organizations might reduce the possibility to interact with a stable group of persons, e.g., teachers and peers, resulting in a lower opportunity to build up good relationships with teachers and peers. In addition, one context might not be able to fulfill the individual needs of some students and, therefore, students might interact with the other context more often to compensate for the lack of integration.

### Social Integration and Intrinsic Motivation: A Socio-Ecological Perspective

The influence of social integration profiles on individual trajectories of intrinsic motivation can be explained through self-determination theory (Ryan and Deci, 2000b), embedded in a socio-ecological perspective (Bronfenbrenner and Morris, 2006). The self-determination theory explains how the fulfillment of three basic needs can facilitate the development of intrinsic motivation: the need for autonomy, competence, and relatedness (Deci and Ryan, 1985). To explain the influence of social integration, we focus on the need for relatedness, e.g., being part of a group (Ryan and Deci, 2000b). The feeling of being integrated fosters positive self-perceptions as a worthy and loved person. Simultaneously, the possibility of participating in academically oriented group interactions (discussions, learning groups) increases, which may provide possibilities to get support from their teachers and peers to solve problems or discuss different perspectives (Ladd et al., 2009). Thus, positive self-perceptions in combination with frequent group interactions might affect the enthusiasm for university studies (Noyens et al., 2019).

As the level of integration regarding teachers and peers can differ resulting in convergent and divergent integration profiles, the socio-ecological theory (Bronfenbrenner and Morris, 2006) might explain the link between different integration profiles and students' development of intrinsic motivation. A key rationale of socio-ecological theory is that a person's development happens in a complex system of social interactions. Interaction partners in the immediate environment who directly interact with the person are categorized as the microsystem. Individuals typically interact with multiple microsystems, for example, peers and teachers at the university. One possibility is that each microsystem uniquely contributes to the development of intrinsic motivation, resulting in additive effects. Previous research provided empirical evidence for additive effects on academic outcomes, e.g., academic achievement, school compliance (e.g., trouble getting homework done), or school identification (e.g., valuing of education; Wang and Eccles, 2012). However, previous research did not focus on intrinsic motivation but on academic outcomes. Because these academic outcomes are related to intrinsic motivation, it seems reasonable to assume that the finding also applies to intrinsic motivation. Concerning this study, teachers and peers can each provide specific support, e.g., through hierarchical or non-hierarchical interactions. Thus, higher integration levels in both systems may additively fulfill the need for relatedness and, in turn, foster a favorable intrinsic motivation development (Ryan and Deci, 2000a, 2020).

Besides such additive effects, the socio-ecological theory explains that microsystems could also interact with each other in mesosystems (Bronfenbrenner and Morris, 2006). Compensatory effects, e.g., the effect of a low integration into one microsystem on the development of intrinsic motivation that is counterbalanced by a high integration into another microsystem, can be argued to represent mesosystemic interactions of the microsystem. Students might focus even more on that well-integrated context to satisfy their need for relatedness, and in turn, might have the same possibility for a favorable development of intrinsic motivation as well-integrated students into both microsystems. Regarding compensatory effects, empirical results do not provide a clear picture. Some studies report that peers may be able to compensate for a bad teacher relationship regarding school engagement, but teachers are not able to compensate for negative peer relationships emphasizing the importance of positive peer relationships (Rosenfeld et al., 2000). Nonetheless, Wang and Eccles (2012) found that social support from teachers could partially counteract the negative influence of peer support on school compliance and school identification.

### This Study

Based on the self-determination theory embedded in a socio-ecological framework, this study focuses on (1) Interindividual differences in university students' developmental trajectories of intrinsic motivation, (2) Interindividual differences in university students' teachers' and peers' integration, and (3) How these patterns are related.

Regarding the development of intrinsic motivation (research question 1), we expect differential trajectories (e.g., decrease, stability, and increase) of intrinsic motivation (Deci and Ryan, 1985).

Concerning students' social peer and teacher integration (research question 2), we expect most students to show convergent integration profiles (for example, High, Medium, and Low for peers and teachers). However, a smaller percentage of persons, who are not integrated in the same way into multiple contexts, is expected. Therefore, there might be divergent integration profiles as well (for example, High, Medium, and Low for peers or teachers).

Bringing both sets of research questions together (research question 3), we assume that if students feel integrated in the same way into both contexts (High, Medium, and Low for peers and teachers), the integration levels might have additive effects on the development of intrinsic motivation. Higher levels of integration into both contexts (teachers *and* peers) are expected to increase the probability of increases in intrinsic motivation or stability on a high level. However, if students are only well-integrated into one context (High, Medium, Low for peers *or* teachers), additive or compensatory effects might be possible. Concerning additive effects, students who are only well-integrated into one context should have a lower probability of favorable trajectories than students on medium or high integration levels into both contexts. Concerning compensatory effects, students who are only well-integrated into one context might have a comparable probability of favorable trajectories as students on medium or high integration levels in both contexts (teachers *and* peers). Thus, the context in which students are better integrated might decrease or even counterbalance the impact of lower integration levels in the other context.

This study contributes to the existing literature in several regards. First, although the underlying theories claim general processes across developmental stages, the researchers mainly focused on adolescents. University students were less focused neglecting the specific environment and the respective interaction partners, like teachers and peers. Second, the study follows a strictly person-centered approach (e.g., Laursen and Hoff, 2006; Xie et al., 2020) focusing on individual trajectories of intrinsic motivation (research question 1), individual profiles of social integration levels in regards to teachers and peers (research question 2) and how they are related (research question 3). Variable-centered studies on the development of intrinsic motivation predominantly apply linear trend models (e.g., linear regression), which might not adequately reflect differential intraindividual change trajectories (Otis et al., 2005). Concerning social integration at the university, variable-centered approaches might focus on general integration levels in both contexts across all students, neglecting heterogeneity in the integration patterns (convergent and divergent profiles). Thus, variable-centered studies might also mask how different levels of social integration into teacher and peer contexts (convergent and divergent profiles) affect individual developmental trajectories of intrinsic motivation (decrease, stability, and increase). Especially, the compensatory effects of divergent profiles are likely to be overlooked. Third, we focused on university students in the middle of their bachelor's studies. Studies provide evidence that for most students, the intrinsic motivation increases during the transition to the university (Ratelle et al., 2004; Kyndt et al., 2015). However, after the first year of bachelor's studies, a critical turnaround occurs, showing that for most of the students, their intrinsic motivation decreased (Brahm and Gebhardt, 2011; Pan and Gauvain, 2012). Therefore, it is important to identify potential developmental pathways during the bachelor's studies and how they can be influenced by students' social peer and teacher integration. Finally, the sample of bachelor's students was not restricted to a specific degree program. Therefore, more generalized conclusions about students' university careers are possible.

## Materials and Methods

### Participants and Procedure

This study used data from the National Educational Panel Study (NEPS) provided by the Research Data Center of the Leibniz Institute for Educational Trajectories (FDZ-LIfBi). NEPS is a large German longitudinal study with multiple cohorts on educational processes and competence development by using multiple data collection methods, such as computer-assisted web interviews (CAWI; Weiß, 2012; Wei and Weber, 2013) and computer-assisted telephone interviewing (CATI; Brachem et al., 2019).

For this study, we used data from the NEPS *Starting Cohort 5—First-Year Students* who enrolled in higher education in Germany for the first time in the winter semester 2010/2011 (further details; Blossfeld and Maurice, 2011).^1^ Capturing the development of student motivation after the first year of their bachelor's studies (Brahm and Gebhardt, 2011; Pan and Gauvain, 2012), time point T1 (third semester, winter semester 2011/2012), and T2 (fifth semester of their studies; 1-year time-lag) of the CAWI survey were chosen. To be able to make valid statements about the development of intrinsic motivation of undergraduate students in Germany, strict criteria were chosen for the formation of the core sample. First, bachelor's students were chosen who did not drop or did not participate in the second wave (CAWI time point 1; *n* = 12,072). In the second step, it was ensured that these students were still enrolled in their higher education in the fifth semester (CAWI time point 2; *n* = 9,071).^2^ In the third step, only those students were included in the core sample who had not changed their subject (*n* = 7,711) because subject changes might also influence students' social integration and the development of intrinsic motivation (Heublein et al., 2010). Moreover, those students who did not provide any information regarding the variables of interest were excluded (*n* = 92). This resulted in a longitudinal core sample of *n* = 7,619 German students.^3^

According to the gender distribution of the NEPS basic sample, in this sample, 36.7 % were males and 63.3 % were female students with an average age of 22.2 (*SD* = 3.64) at the T1. Most of the students were enrolled in language and cultural studies (26.5 %), followed by 25.4% in law/economics/social science and mathematics and natural science with 21.6, 13.9% engineering, 6.1% human medicine/health science, 2.7% arts/aesthetics, 2.3 % agricultural/forest/and nutrition, 1.3% sports, and 0.3% were enrolled in veterinary medicine. This distribution of subject groups largely corresponds to the distribution in the population (Statistisches Bundesamt, 2011).

### Measures

The instruments used in this article are overall constructed by NEPS and are explained in more detail below (the list of variables representing the constructs of interest is shown in Supplementary Table 1).

#### Intrinsic Motivation

Three items of the Academic Commitment Scale by Grässmann et al. (1998) served as indicators (e.g., “I really enjoy my degree course”). The items were rated on a five-point rating scale (“does not apply at all” (1) to “does completely apply” (5)) and showed good reliability at both measurement occasions (Cronbach's α time point 1 = 0.84 and Cronbach's α time point 2 = 0.86).

#### Social Integration

According to Dahm et al. (2016), social integration regarding peers was assessed with three items by Schiefele et al. (2002); (e.g., “I have been successful in building contacts with other students during my studies up to now.”). As there is no established German scale for social integration regarding teachers, four additional items from different origins were put together covering this aspect (e.g.; “I feel accepted by the instructors.”). Two items were from SACQ (Baker and Siryk, 1999), one item from Wosnitza (2007), and one item was an internal NEPS development adapted from PISA (Hertel et al., 2010). All items on these two constructs were rated on four-point scales (“does not apply at all” (1) to “does completely apply” (4)). Both showed a good reliability (Cronbach's α peers = 0.84 and Cronbach's α. teachers = 0.75).

#### Control Variable

Degree program (dummy coded, reference category = language and cultural studies) was included as a control variable to secure valid interpretations of our research questions across all degree programs. As each of the degree programs has different access criteria as well as a different number of students, both might be influential for the development of intrinsic motivation as well as the possibilities of social integration. Further control variables, like age and gender, were not included due to their low effect sizes (Gillet et al., 2009; Schaeper, 2020).

### Analyses

#### Preliminary Analyses

The result of a confirmatory factor analysis supported the two-factor structure of peer and teacher social integration, χ^2^ (13, *n* = 7619) = 180.52, *p* < 0.001, RMSEA = 0.04, SRMR = 0.02, CFI = 0.99, TLI = 0.98.

To secure the interpretation of changes in the dependent variable over time (Schmitt et al., 2011), the latent intrinsic motivation variables were tested for their invariance across time along three steps (Cheung and Rensvold, 2002; Chen, 2007). The first step tested the configural invariance, modeling intrinsic motivation with the same set of items at both measurement occasions. This model showed a good fit, χ^2^ (5, *n* = 7619) = 42.18, *p* < 0.001, RMSEA = 0.03, SRMR = 0.01, CFI = 1.00, TLI = 1.00, indicating configural invariance. The second step tested metric invariance by restricting the factor loadings to be equal across time points. The comparison between the configural and the metric model showed no systematic difference, ΔCFI = 0.001 and ΔRMSEA = 0.009, indicating metric invariance since the CFI difference is smaller than 0.01 and the RMSEA difference is smaller than 0.015 (see, Cheung and Rensvold, 2002; Chen, 2007). The third step tested scalar invariance by additionally fixing the manifest intercepts to be equal. The difference test showed that the additional constraints of the intercepts showed an ambivalent picture, ΔCFI = 0.003 and ΔRMSEA = 0.035, indicating that not all indices showed no systematic difference. Therefore, we tested for partial scalar invariance (Brown, 2006) constraining only two intercepts to be equal. This model did not differ from the metric model, ΔCFI = 0.000 and ΔRMSEA = 0.000, indicating partially invariant constructs over time for latent change models (for a detailed discussion of partial invariance see Brown, 2006).

#### True-Intraindividual-Change Mixture Model (TIC-MM) and Latent Profile Analysis (LPA)

True-Intraindividual-Change Mixture Models (TIC-MM) and Latent Profile Analysis (LPA) were used to identify subgroups of students with different profiles of intrinsic motivation development (TIC-MM) as well as social integration profiles (LPA).

For the investigation of intraindividual changes in students' intrinsic motivation, we specified a True-Intraindividual-Change (TIC) Model (Steyer et al., 1997). In detail, a latent intercept depicting the students' time point 1 value on the respective variable and a latent change variable were specified. Following the idea of person-centered analyses, in the next step, we tested if there are homogenous subgroups of students sharing comparable developmental patterns (adding the mixture part). Thus, the intrinsic motivation factor scores^4^ for the intercept and the change of intrinsic motivation obtained from the TIC models were administered to mixture analyses, testing research questions 1. In these analyses, a three-step approach was used (Pastor et al., 2007), implemented in Mplus 8 (Muthén and Muthén, 2017): (1) Identifying a profile solution, (2) Examining the classification accuracy, and (3) Analyzing the relationship between profile membership and covariates (college degree program) identifying potential predictors for profile memberships. The decision for the final profile solution was driven by theoretical considerations as well as by the fit indices of the specified models (Nylund et al., 2007; Geiser, 2011). Commonly used information criteria are the AIC (Akaike), the BIC, and the adjusted BIC, and finding the lowest values on these three criteria indicates the best profile solution. The Lo-Mendell-Rubin Likelihood Ratio-Test (Lo et al., 2001), implemented in Mplus served as a significance test to compare the class solutions. This test compares a *k* solution of clusters with a *k-1* solution. If there is no significant improvement between the two solutions, the k-1 solution should be chosen. One problem with these tests is the dependency on sample size (Marsh et al., 2009). Additional information can be elbow plots. The elbow of the curve indicates the number of profiles to select and illustrates the benefit of an additional profile (Gillet et al., 2017). Taking these criteria into account, the researcher's decision about the number of profiles is more informed compared to traditional cluster analysis (Pastor et al., 2007; Marsh et al., 2009).

For the second step, after we decided in favor of a profile solution, the average posterior class probability (AvePPk) and the entropy allowed a separate decision on the goodness for every class. AvePPk range between 0 and 1, “1” representing a perfect classification for all individuals within this class. A criterion value higher than 0.70 denotes a good classification (Nagin, 2005; Masyn, 2013). The entropy ranges between 0 and 1 with higher values indicating a better classification utility (Pastor et al., 2007).

The covariate degree program was included in the third step. If the covariate has a substantial influence on the profile memberships, further analyses were calculated based on the results, where the covariate was included.

For identifying subgroups of students characterized by different patterns of social integration, the standardized factor scores^5^ of the social integration scales (peers and teachers) were entered in the Latent Profile Analysis (LPA). The rationale and steps are parallel to those described above.

#### Latent Transition Analysis

The final profile solutions of the LPA and the TIC-MM were combined into the latent transition analysis. We used the class assignment (logits) from the final LPA and TIC-MM predicting the profiles of intrinsic motivation through the social integration profiles. This procedure ensured that the class assignment proves stable (Nylund-Gibson et al., 2014).

## Results

The results are divided into three sections according to the research questions. First, we report the findings of the Latent Profile Analysis for the development of intrinsic motivation (research question 1) and the social integration profiles (research question 2). In a final step, we combined the two models in testing our last research question (research question 3).

### TIC-MM Intrinsic Motivation Development

The True-Intraindividual-Change Mixture analyses revealed that the three-profile solution for the development of intrinsic motivation provided the best fit to the data (see Table 1). In detail, all fit indices considerably improved up to the three-profile solution. Adding a fourth profile, the fit indices decreased to a smaller extent (see elbow plots in the Supplementary Figure 1). Therefore, we chose the three-profile solution. The entropy was 0.73 and the AvePPk for each profile was above 0.73, indicating a good classification utility (see Table 2).

**Table 1:** Fit indices for TIC-MM; intrinsic motivation.

**Number of classes**	**1**	**2**	**3**	**4**
**Number of free parameters**	**5**	**10**	**15**	**20**
AIC	23,749.109	22,195.919	20,971.894	20,625.900
BIC	23,783.801	22,265.303	21,075.970	20,764.668
ABIC	23,767.912	22,233.526	21,028.303	20,701.112
LO-Mendell	NA	0.00	0.00	0.00
Entropy	NA	0.969	0.730	0.595

*AIC, Akaike Information Criterion; BIC, Bayesian Information Criterion; ABIC, Adjusted BIC; NA, Test is not available for one-class-model*.

**Table 2:** Average latent class probabilities for most likely latent class membership (row) by latent class (column).

**Profile**	***n***	**1**	**2**	**3**
Increase	532	0.943	0.013	0.045
Moderate decrease	2,580	0.000	0.735	0.265
Decrease	4,507	0.001	0.069	0.930

**Figure 1:**
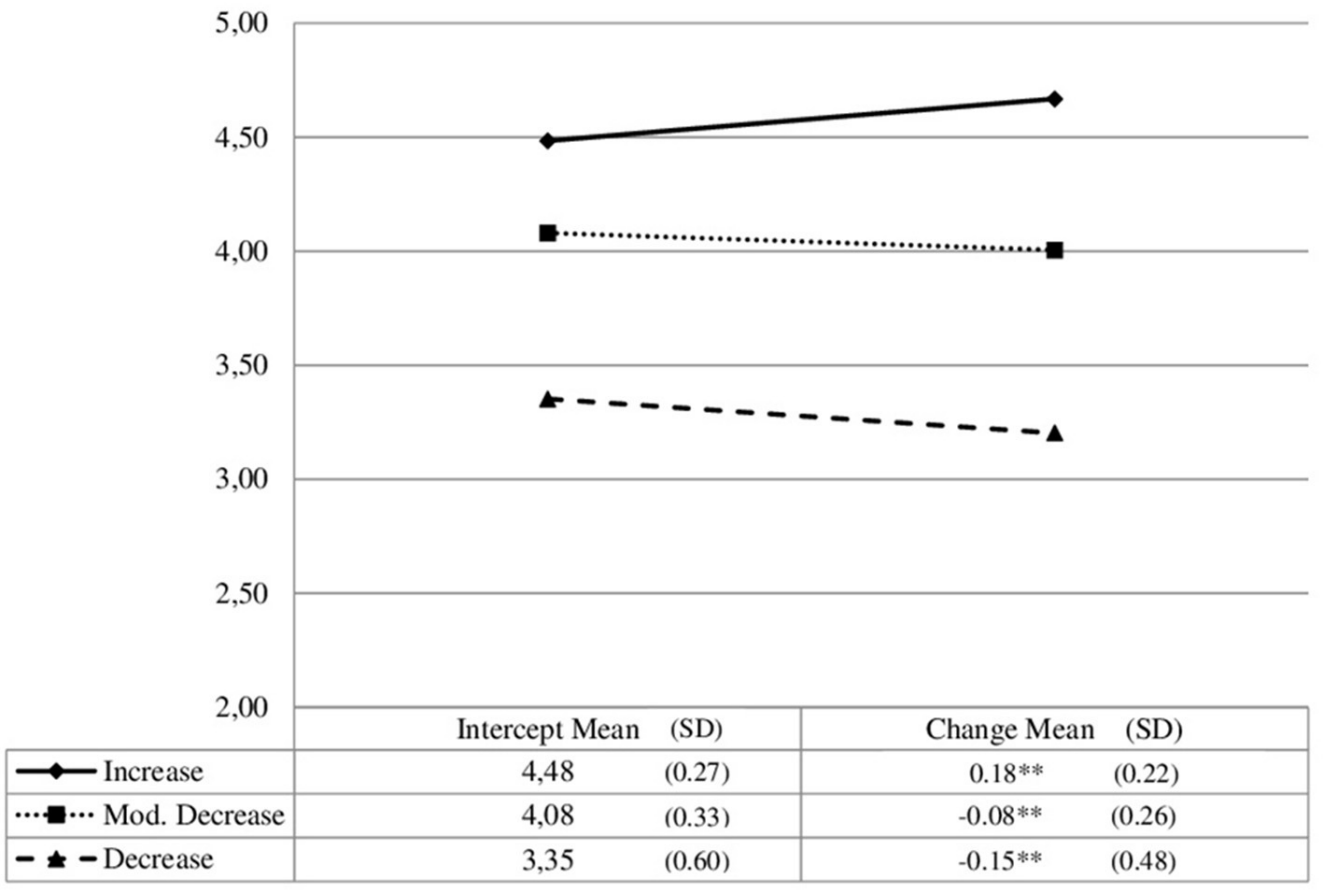
Development of intrinsic motivation (raw scores means). ^**^p < 0.01.

In line with our first research question, students can be grouped into three profiles for the development of intrinsic motivation (see Figure 1). The first profile comprised 7% of the students. Students in this profile are characterized by a high level and even significant increases in intrinsic motivation over 1 year (*Increase)*. In the second profile, students (33.9%) reported a moderate level of intrinsic motivation with only a slightly significant decrease at this level (*Moderate Decrease)*. Most of the students (59.2%) were grouped into a third profile, with low levels of intrinsic motivation and a significant decrease over time (*Decrease)*. The inclusion of the covariate college major showed significant predictions in the profiles (see elbow plots in the Supplementary Table 2). Therefore, we included this covariate for further analysis.

### LPA Social Integration

The Latent Profile Analysis for social integration revealed that the four-profile solution provided the best fit for the data (see Table 3). In detail, all fit indices considerably improved up to the fourth profile solution. Adding a fifth profile, the fit indices improved only to a smaller extent indicating that an additional fifth profile did not add explanatory value (see Supplementary Figure 2). Therefore, we decided to choose the four-profile solution. The entropy was 0.89 and the AvePPk for each profile was above 0.88, indicating a good classification utility (see Table 4).

**Table 3:** Fit indices for LPA social integration.

**Number of**	**1**	**2**	**3**	**4**	**5**
**classes**					
**Number of free**	**4**	**7**	**10**	**13**	**16**
**parameters**					
AIC	43,263.343	42,382.653	41,162.521	40,420.787	40,170.837
BIC	43,291.097	42,431.222	41,231.905	40,510.987	40,281.851
ABIC	43,278.386	42,408.977	41,200.127	40,469.675	40,231.006
LO-Mendell	NA	0.000	0.000	0.000	0.000
Entropy	NA	0.582	0.854	0.898	0.890

*AIC, Akaike Information Criterion; BIC, Bayesian Information Criterion; ABIC, Adjusted BIC; NA, Test is not available for one-class-model*.

**Table 4:** Average latent class probabilities for most likely latent class membership (row) by latent class (column).

**Profile**	***n***	**1**	**2**	**3**	**4**
Highly integrated	2,492	0.962	0.038	0.000	0.000
Moderately integrated	3,832	0.042	0.925	0.032	0.000
Isolated	1,144	0.047	0.000	0.945	0.007
Peer deprivated	151	0.000	0.000	0.114	0.886

**Figure 2:**
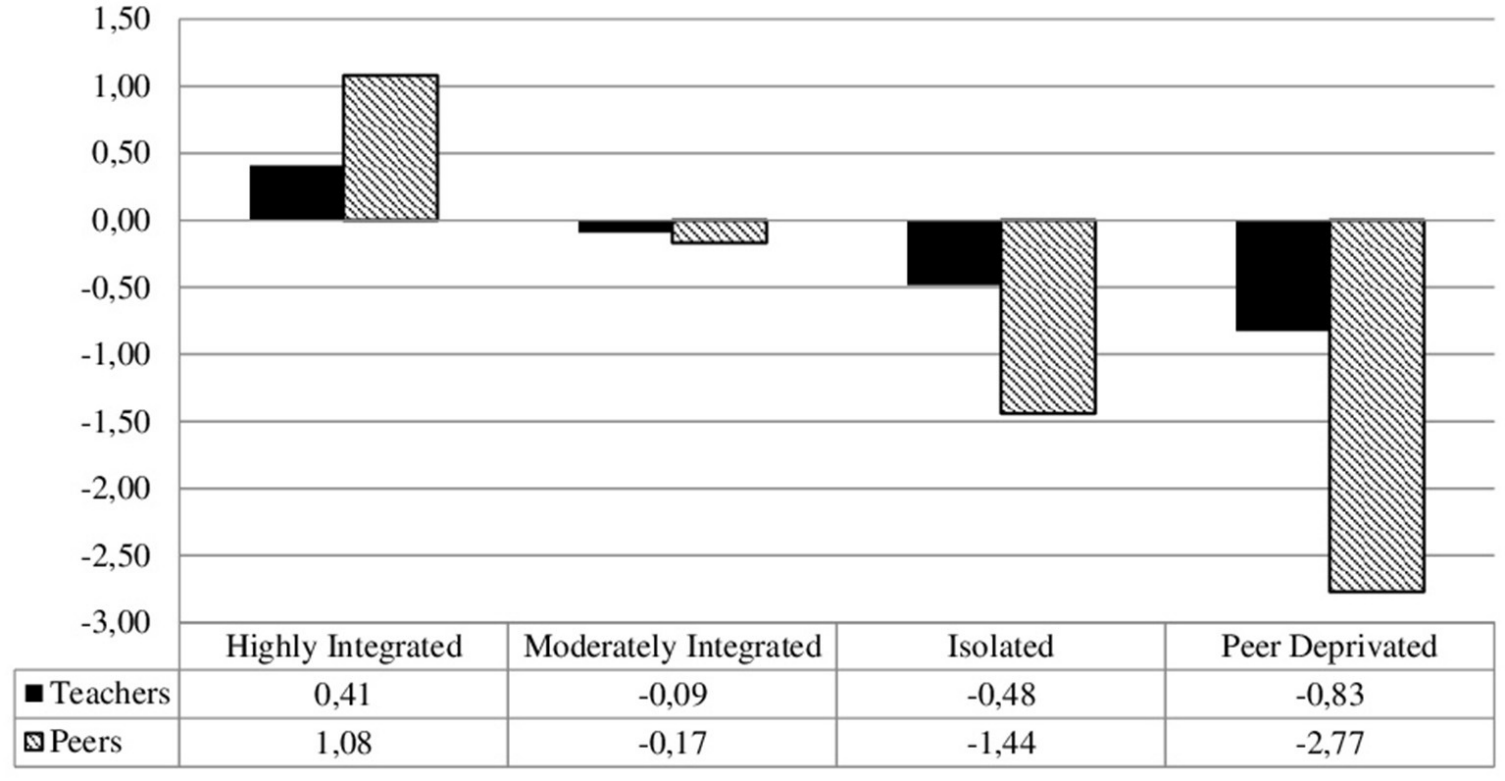
Social integration: teachers and peers (z-standardized means). The integration profiles were calculated based on the means: SD within each profile are for teacher integration (SD = 0.89) and peer integration (SD = 0.12).

In line with our research question, three convergent integration profiles were identified (see Figure 2). The first profile, which comprised 32.7% of the students, was characterized by the highest levels of peer and teacher integration above the average compared to the other profiles (*Highly Integrated*). The second profile consisted of most of the students (50.3%). Typical for this profile were average scores of peer and teacher integration (*Moderately Integrated*). In the third profile (15.0% of the students), peer and teacher integration was below the average (*Isolated*). In the fourth profile (2.0% of the students), a combination of extremely low peer integration with relatively low teacher integration was found (*Peer Deprivated*). Because this is the only profile in which the difference between peer and teacher integration exceeds one *SD*, this profile can be classified as a divergent profile.

The inclusion of the covariate college major did not cause substantial changes in the profile solution. Therefore, we excluded this covariate for further analysis in the prediction of social integration profiles (see Supplementary Table 3).

### Latent Transition Analysis

The transition analysis (see Table 5) revealed that most of the students of each integration profile can be found in the *Decrease* profile. Nevertheless, the students of the *Highly Integrated* profile showed the lowest probability (47.2%) that their intrinsic motivation *decreased*, followed by students of the *Moderately Integrated* profile with the probability of 67.8%. Students of the *Isolated* (86.8%) and the *Peer Deprivated* (91.7%) profiles had nearly the same, and compared to the other profiles, the highest probability for a *decrease* in their intrinsic motivation.

**Table 5:** Latent transition probabilities based on the estimated model.

	**Intrinsic motivation**		
**Social integration**	**Increase** **(*n* = 532)**	**Moderate Decrease** **(*n* = 2,580)**	**Decrease** **(*n* = 4,507)**
Highly integrated (*n* = 2,492)	0.114	0.414	0.472
Moderately integrated (*n* = 3,832)	0.046	0.276	0.678
Isolated (*n* = 1,144)	0.030	0.102	0.868
Peer deprivated (*n* = 151)	0.027	0.056	0.917

Students of each integration profile showed a lower probability of the *Moderate Decrease* profile than the *Decrease* profile. However, students of the *Highly Integrated* profile were more likely to *moderately decrease* in their intrinsic motivation (41.4%), than students of the *Moderately Integrated* profile with 27.6%. Moreover, only 5.6% of the students of the *Peer Deprivated* and 10.2% of the students of the *Isolated* profile were found in the *Moderate Decrease* profile.

Finally, the results revealed that students generally showed the lowest probability for the *Increase* profile compared to the *Moderate Decrease* and *Decrease* profiles. However, students of the *Highly Integrated* profile had the highest probability to *increase* their intrinsic motivation (11.4%), followed by students of the *Moderately Integrated* profile (4.6%), the *Peer Deprivated* profile (2.7%), and the *Isolated* profiles (3.0%) with nearly the same transition probabilities.

## Discussion

The results of this study provide evidence that the focus on person-centered approaches (e.g., Laursen and Hoff, 2006; Xie et al., 2020) give substantial insights into individual pathways of intrinsic motivation (*Increase, Moderate Decrease*, and *Decrease*), individual social integration patterns (convergent, divergent), and in turn, into additive and compensatory effects of the integration profiles. First, our study provided evidence for three profiles regarding students' development of intrinsic motivation. Comparable to earlier variable-centered results (Müller and Palekčić, 2005; Brahm and Gebhardt, 2011; Pan and Gauvain, 2012; Bieg et al., 2017), most of the students reported a decrease or moderate decrease in intrinsic motivation (93.1%) during 1 year. However, the results also revealed a small percentage of students (7%) with increasing intrinsic motivation. This percentage is quite similar to person-centered results found by Gillet et al. (2017) for psychology students within one semester. Concerning previous results (Ratelle et al., 2004; Kyndt et al., 2015), which showed that in the first year of bachelor's studies, intrinsic motivation increased for most of the students, it could be of interest as to when and under which conditions this increase changed into a decrease for most of the students.

Second, we found four profiles of social integration. In line with theoretical rationales (Bretherton, 1995) and earlier empirical findings (Ciarrochi et al., 2017), most of the students can be grouped into three convergent profiles (*Integrated, Moderately Integrated, and Isolated*). However, a smaller percentage of students showed a profile that is characterized by an extremely low integration into the peer context and a low integration into the teacher context (*Peer Deprivated*). We classified the *Peer Deprivated* profile as divergent. But in contrast to our assumptions, we did not find any divergent profiles where only teacher or peer integration was high (*Peer Integrated or Teacher Integrated*). In this regard, students in the third semester may be settled at the university (Coertjens et al., 2017) and build up relationships with teachers and peers in the same way. Therefore, divergent integration profiles should be more likely in the beginning of university studies when relationships were built-up (Pan and Gauvain, 2012). Another explanation might be based on the Halo-effect (Forgas and Laham, 2017) that within the convergent profiles some students may generalize the level of integration between contexts and therefore reduce the levels of ambivalent information. In this regard, we would assume that students generalize the level of peer integration to some degree onto the level of teacher integration than vice versa. As students may interact with their fellow students more intensively inside as well as outside the university as compared to their teachers, students may perceive the integration into the peer context as more differentiated than the integration with teachers (also reflected in a higher range in the peer integration scale compared to the teacher integration scale).

However, for most of the students (*Highly Integrated* and *Moderately Integrated*) an important precondition, such as a good social integration, for a favorable development of intrinsic motivation should be fulfilled at least to some extent. This is in line with studies on adolescents showing that most students can be assigned to profiles where they feel at least moderately supported (Ciarrochi et al., 2017). Even though it is a smaller group, students of the *Isolated* and *Peer Deprivated* profiles, emphasis should be placed particularly on the importance of social integration in these two contexts (see Practical implications), especially because social integration is not only important for the development of intrinsic motivation but also for student well-being (Taylor, 2011; Awang et al., 2014).

The more integrated students are in both contexts, the higher the probability that their motivation decreases only slightly or even increases. Thus, additive effects (the more the better) can be assumed for the influence of peer and teacher integration as long as the integration stays above average. Thus, good integration into both contexts might explain that students focus on their university studies with enthusiasm because they feel integrated and supported by others. Although most of the students of the *Highly Integrated* profile were found in the *Increase* or *Moderate Decrease* profile (in sum 52.8%), indicating that their motivation stayed at least on a moderate level, 47.2% of those students were found in the *Decrease* profile. One explanation for the high probability of an unfavorable development of intrinsic motivation might also be found in a high peer integration. In detail, if students are highly integrated into a peer context with low levels of academic motivation, students might get more distracted than motivated for their university studies. This is supported by studies on adolescents showing that high peer integration might not always have positive effects on student development, for example, their aggressive behavior (for summary; Juvonen, 2006). Thus, it could be fruitful for future studies to include the respective peer characteristics to get a better idea of why students' change in the decrease profile despite a high or moderate integration into this context exists.

If the teacher and peer integration was below average (*Isolated and Peer Deprivated*), the probabilities for intrinsic motivation profiles were nearly the same, e.g., the intrinsic motivation of most of the students decreased over 1 year. This effect pattern extends the previous variable-centered results indicating linear effects of relatedness on intrinsic motivation (Capon-Sieber et al., 2022). There might be two possible explanations for this effect pattern. First, based on threshold models, it could be assumed that if peer and teacher integration are below a critical point indicating that students feel poorly integrated into both contexts, students' motivation decreases independently of the level of integration. Therefore, an additive effect (the less the weaker) of teacher and peer integration cannot be determined when a critical point of integration into both contexts is reached. Second, indications for a compensatory effect can be found when comparing the transition probabilities of the *Peer Deprivated* and *Isolated* profiles. Both have the same transition probabilities but the *Peer Deprivated* profile showed an extremely low peer integration and a higher teacher integration, whereas the *Isolated* profile showed a higher integration regarding teachers and peers than the *Peer Deprivated* profile. Therefore, the higher teacher integration might have counterbalanced the low peer integration effects in the *Peer Deprivated* profile. This result might be in line with empirical results by Wang and Eccles (2012) where teachers might compensate to some degree on a low peer integration.

### Practical Implications

The results showed that 17% of the students are integrated below the sample average into both contexts (*Isolated* or *Peer Deprivated* profile). This percentage is in line with results that students feel lonely throughout their first semester (e.g., Wiseman et al., 2006). Based on the assumption of a threshold model, universities are in charge to establish formats to integrate the low integrated students (*Isolated* or *Peer Deprivated* profile) equally increasing the probability that those students feel connected to the university, and in turn, that their intrinsic motivation stays at least on a high level.

However, to implement appropriate integration initiatives, both (1) personal and (2) environmental factors have to be considered (Schaeper, 2020). Person-related factors are necessary to better characterize the group of poorly integrated people, e.g., as in the *Isolated or Peer Deprivated* profiles. One of these factors is the socio-demographic or cultural background. Linguistic and cultural differences can represent potential barriers to social integration. For an initial appraisal, we also tested the distribution of student migration backgrounds between the profiles. The results did not show any significant differences in terms of the migration background between the four integration profiles with a quite similar distribution of ~20% of students with a migration background per profile. Nonetheless, future research is still required to identify person-related factors that are responsible that some students are better integrated than others despite their migration background. Another factor could be the employment of students parallel to their studies. Students from educationally disadvantaged families with low incomes may have problems financing their studies because they receive less financial support from their parents and are dependent on gainful employment (Heublein et al., 2010). Thus, the financial pressure makes it difficult for many students to recognize their studies as their main activity, which means that less time can be spent on campus and with fellow students due to working hours.

Integration-friendly environments can be created through activities, such as sports events, communication workshops, or culture-related festivals (Owens and Loomes, 2010). As social integration is essential for both national and international students, these meeting areas might be also a possibility for integrating and connecting people and therefore enhance students' social integration on- and off-campus and minimize language and cultural barriers. Furthermore, senior students could act as assistants and advisers for younger students and support them in learning and preparing tests and homework. This enables the exchange of experience with older students and not only promotes social integration but may also, in the long run, increase the students' intrinsic motivation through successful examinations (Baars and Arnold, 2014).

### Limitations

There are some limitations that have to be kept in mind when interpreting the results. First, quite a high number of students were excluded from the core sample due to their dropout from university studies or changing the subject. The results of additional analyses revealed that those excluded students scored lower on all variables of interest at the first time point (third semester). Therefore, we would assume that these students are more likely to be found in the unfavorable social integration profiles potentially influencing the probabilities of the *Isolated* and *Peer Deprivated* profiles in this study. Second, female students are overrepresented in the sample (due to the oversampling of teacher students to some degree). This bias is a general problem in online surveys (e.g., Smith, 2008; Saleh and Bista, 2017). Possibilities for future surveys might be to send additional email reminders that showed positive effects on the participation rate of male students (Saleh and Bista, 2017). Third, although some personal characteristics explain low variance in social integration as well as intrinsic motivation (Gillet et al., 2009; Schaeper, 2020), future studies should also include personal characteristics, such as personal motives for studying and/or choosing a college major to combine personal preconditions with conditions in the environment, such as integration into peer and teacher context to predict the development of intrinsic motivation in greater detail. At least, the results are limited to a specific student cohort of NEPS from 2010 to 2016. Therefore, the results of this study cannot be completely transferred to the current student cohort. There might be social changes that affect student interactions with teachers and peers and in turn the results of this study. Therefore, the results should be validated by students who are currently studying.

### Summary

Despite these limitations, this study contributes to the literature in several regards. First, the results of the study revealed that university students of all degree programs showed differential developmental trajectories of intrinsic motivation during 1 year. Second, the results showed that students can be grouped into different integration profiles and those profiles are mostly convergent profiles. Third, this study indicates that integration below the average is linked to negative developmental trajectories independent of the extent of poor integration.

## Data Availability Statement

Publicly available datasets were analyzed in this study. This data can be found here: https://www.neps-data.de/Datenzentrum/Daten-und-Dokumentation/Startkohorte-Studierende.

## Ethics Statement

Ethical review and approval was not required for the current study in accordance with the local legislation and institutional requirements. Written informed consent for participation was also not required for the current study in accordance with the national legislation and the institutional requirements. Ethical approval was not required for the NEPS study that produced the data on which the current study is based because the NEPS study was conducted under the supervision of the German Federal Commissioner for Data Protection and Freedom of Information (BfDI) and in coordination with the German Standing Conference of the Ministers of Education and Cultural Affairs (KMK) and—in the case of surveys at schools—the Educational Ministries of the respective Federal States. All data collection procedures, instruments and documents were checked by the data protection unit of the Leibniz Institute for Educational Trajectories (LIfBi). The necessary steps were taken to protect participants' confidentiality according to national and international regulations of data security. Participation in the NEPS study was voluntary and based on the written informed consent of participants. This consent to participate in the NEPS study can be revoked at any time.

## Author Contributions

MR and TA made substantial contributions to the conception of the work, analysis and interpretation of data for the work, and drafted the work. BG revised it critically for important intellectual content. All authors contributed to the article and approved the submitted version.

## Funding

The open access publication fee was funded by the Paris-Lodron University of Salzburg.

## Conflict of Interest

The authors declare that the research was conducted in the absence of any commercial or financial relationships that could be construed as a potential conflict of interest.

## Publisher's Note

All claims expressed in this article are solely those of the authors and do not necessarily represent those of their affiliated organizations, or those of the publisher, the editors and the reviewers. Any product that may be evaluated in this article, or claim that may be made by its manufacturer, is not guaranteed or endorsed by the publisher.

## Footnotes

^1^Participation was voluntary and the target person's declaration of consent was mandatory for the recruitment process.

^2^With a drop-out rate of 24%, the attrition between the first and second measurement time corresponds to the general drop-out rate in the Bachelor's degree at German universities in the graduate years 2012-2014 with 28%.

^3^Several t-tests were calculated comparing the variables of interest between students who dropped out and students who remained at the study. The results revealed that students who dropped out differed regarding their peer integration, *T* (11 433) = 8.83, *p* = .00, teacher integration, *T* (8095) = 5.33, *p* = .00, and intrinsic motivation, *T* (7725) = 9.56, *p* = .00.

^4^The advantage of factor scores is that they provide a partial control for measurement errors in weighting items (Gillet et al., 2017), in cases the full latent model cannot be administered to the mixture analyses because of the model complexity.

^5^Standardization ensured a comparable interpretation of the scale scores.
