# Supplementary material for: Social Integration in Higher Education and Development of Intrinsic Motivation: A Latent Transition Analysis
Source: Front Psychol. 2022 Jun 14;13:877072. doi: 10.3389/fpsyg.2022.877072 (PMC9239343; doi:10.3389/fpsyg.2022.877072)
Supplement: Supplementary file 1 [file Data_Sheet_1.PDF]

Electronic Supplementary Material

Table S-1 | Overview of the measures.

Variable Name	*Items*
	Intrinsic Motivation
tg53232	"I really enjoy my degree course" (Enjoyment of degree program)
tg53236	"I can fully identify with my degree program" (Identification)
tg53234	"My degree program is not really all that fun" (Don't enjoy degree course very much) (-)
	Social Integration: Teachers
tg53111	"I feel accepted by the instructors." (Acceptance by instructors)
tg53112	"I get along well with the instructors in my degree program." (Get along well with instructors)
tg53113	"Most of the instructors treat me fairly." (Fair treatment from instructors)
tg53114	"The lecturers are interested in what I have to say." (Instructors interested)
	Social Integration: Peers
tg53121	"I have been successful in building contacts with other students during my studies up to now." (Able to establish contacts with students)
tg53122	"I know a lot of classmates with whom I can exchange ideas about questions in my field of study." (Speak with classmates about questions in field of study)
tg53123	"I have many contacts with students in my class." (Many contacts with students)

Note: (-) reverse coded

Table S-2 | Covariate (major degree) results for intrinsic motivation profiles.

Profile	Effect	Logit	S.E.	Logit/S.E.	p Value
Decrease	Sports	0.201	0.472	0.427	0.669
	Law, economics and social science	0.135	0.150	0.902	0.367
	Mathematics, natural sciences	0.369	0.166	2.220	0.026
	Human medicine/health sciences	-0.146	0.199	-0.733	0.464
	Veterinary medicine	1.138	1.161	0.980	0.327
	Agricultural-, forest- and nutrition sciences	-0.130	0.322	-0.405	0.685
	Engineering	0.406	0.193	2.109	0.035
	Arts, aestethics	-0.090	0.254	-0.355	0.722
Moderate Decrease	Sports	0.105	0.439	0.238	0.811
	Law, economics and social science	0.378	0.135	2.791	0.005
	Mathematics, natural sciences	0.604	0.152	3.980	0.000
	Human medicine/health sciences	-0.469	0.185	-2.529	0.011
	Veterinary medicine	0.429	1.165	0.368	0.713
	Agricultural-, forest- and nutrition sciences	-0.222	0.293	-0.759	0.448
	Engineering	0.580	0.177	3.274	0.001
	Arts, aestethics	-0.864	0.254	-3.395	0.001

Note. The *Increase* profile is the reference category.

Table S-3 | Covariate (major degree) results for social integration profiles.

Profile	Effect	Logit	S.E.	Logit/S.E.	p Value
Moderately Integrated	Sports	-0.159	0.343	-0.464	0.643
	Law, economics and social science	-0.003	0.101	-0.029	0.977
	Mathematics, natural sciences	0.222	0.110	2.012	0.044
	Human medicine/health sciences	0.064	0.176	0.364	0.716
	Veterinary medicine	0.389	0.712	0.547	0.584
	Agricultural-, forest- and nutrition sciences	0.543	0.293	1.855	0.064
	Engineering	0.170	0.122	1.394	0.163
	Arts, aestethics	0.325	0.268	1.209	0.227
Highly Integrated	Sports	0.245	0.324	0.757	0.449
	Law, economics and social science	-0.136	0.102	-1.331	0.183
	Mathematics, natural sciences	0.237	0.110	2.146	0.032
	Human medicine/health sciences	0.438	0.169	2.596	0.009
	Veterinary medicine	0.154	0.736	0.210	0.834
	Agricultural-, forest- and nutrition sciences	0.403	0.297	1.357	0.175
	Engineering	-0.012	0.125	-0.097	0.923
	Arts, aestethics	0.609	0.260	2.338	0.019
Peer Deprivated	Sports	-0.923	1.364	-0.677	0.498
	Law, economics and social science	0.116	0.247	0.469	0.639
	Mathematics, natural sciences	-0.079	0.289	-0.273	0.784
	Human medicine/health sciences	-1.352	0.886	-1.525	0.127
	Veterinary medicine	-17.449	0.000	0.000	1.000
	Agricultural-, forest- and nutrition sciences	0.705	0.580	1.216	0.224
	Engineering	-0.638	0.400	-1.598	0.110
	Arts, aestethics	-0.084	0.727	-0.116	0.908

Note. The *Isolated* profile is the reference category.

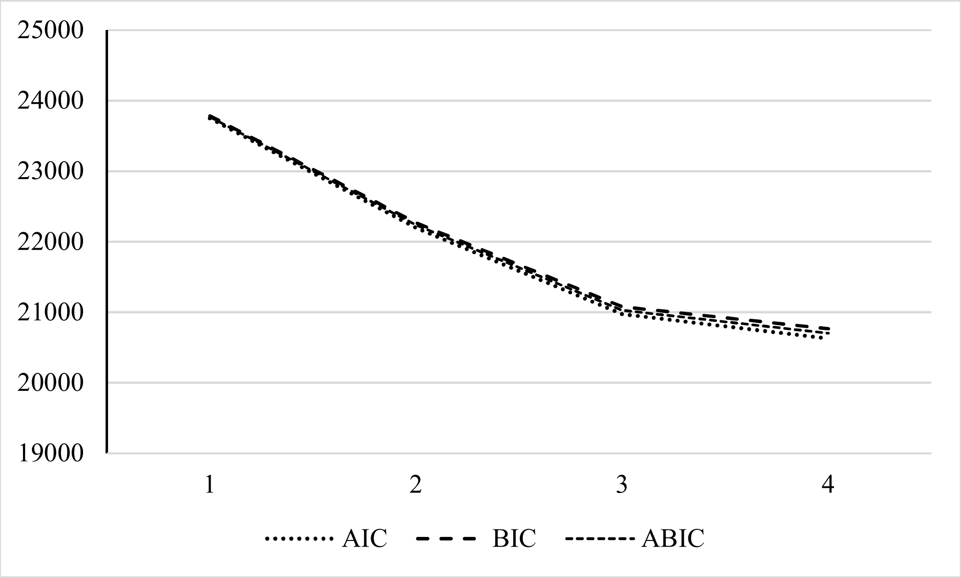

Figure S-1 | Elbow-Plot for class enumeration of intrinsic motivation.

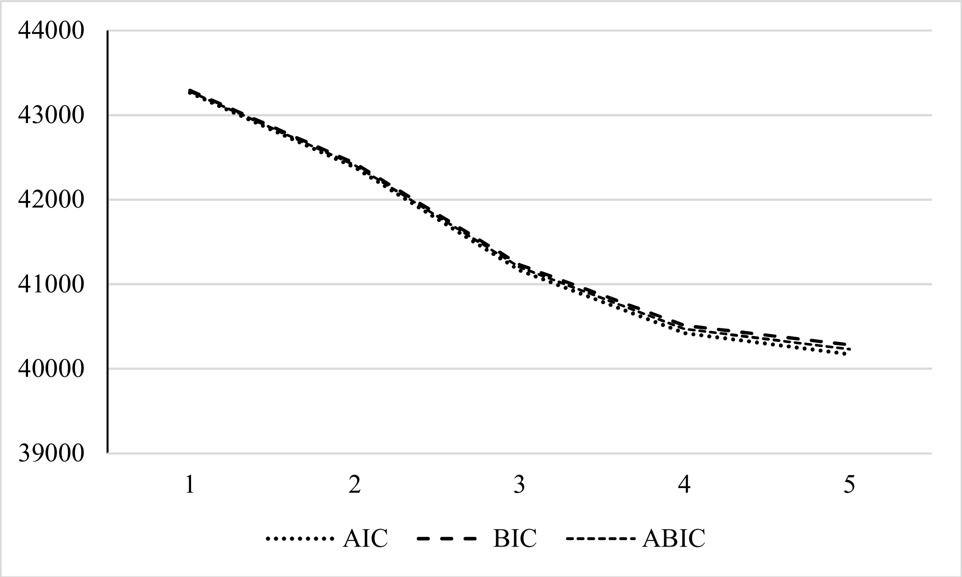

Figure S-2 | Elbow-Plot for class enumeration of social integration.
